# Coactivation of Autonomic and Central Nervous Systems During Processing of Socially Relevant Information in Autism Spectrum Disorder: A Systematic Review

**DOI:** 10.1007/s11065-023-09579-2

**Published:** 2023-02-27

**Authors:** Suvi Karjalainen, Tuija Aro, Tiina Parviainen

**Affiliations:** 1 Department of Psychology, University of Jyväskylä, PO Box 35, FI-40014 Jyväskylä, Finland; 2 Centre for Interdisciplinary Brain Research, University of Jyväskylä, Jyväskylä, Finland

**Keywords:** Autism spectrum disorder, Autonomic nervous system, Central nervous system, Body-brain interaction, Social information processing

## Abstract

Body-brain interaction provides a novel approach to understand neurodevelopmental conditions such as autism spectrum disorder (ASD). In this systematic review, we analyse the empirical evidence regarding coexisting differences in autonomic (ANS) and central nervous system (CNS) responses to social stimuli between individuals with ASD and typically developing individuals. Moreover, we review evidence of deviations in body-brain interaction during processing of socially relevant information in ASD. We conducted systematic literature searches in PubMed, Medline, PsychInfo, PsychArticles, and Cinahl databases (until 12.1.2022). Studies were included if individuals with ASD were compared with typically developing individuals, study design included processing of social information, and ANS and CNS activity were measured simultaneously. Out of 1892 studies identified based on the titles and abstracts, only six fulfilled the eligibility criteria to be included in synthesis. The quality of these studies was assessed using a quality assessment checklist. The results indicated that individuals with ASD demonstrate atypicalities in ANS and CNS signalling which, however, are context dependent. There were also indications for altered contribution of ANS-CNS interaction in processing of social information in ASD. However, the findings must be considered in the context of several limitations, such as small sample sizes and high variability in (neuro)physiological measures. Indeed, the methodological choices varied considerably, calling for a need for unified guidelines to improve the interpretability of results. We summarize the current experimentally supported understanding of the role of socially relevant body-brain interaction in ASD. Furthermore, we propose developments for future studies to improve incremental knowledge building across studies of ANS-CNS interaction involving individuals with ASD.

**Supplementary Information:**

The online version contains supplementary material available at 10.1007/s11065-023-09579-2.

## Introduction

Body-brain interaction provides a novel approach to understand neuropsychiatric and neurodevelopmental conditions, such as autism spectrum disorder (ASD), more comprehensively. ASD is a neurodevelopmental condition characterised by deficits in social communication and interaction along with a variety of restricted repetitive behaviours, focused interests, and activities (American Psychiatric Association, 2013). Although there is a vast amount of literature regarding autonomic and central nervous system (ANS and CNS, respectively) atypicalities among individuals with ASD (e.g., Arora et al., 2021; Hutt et al., 1975; O’Reilly et al., 2017; Palkovitz & Wiesenfeld, 1980; Pardo & Eberhart, 2007), less is known about the interaction between ANS and CNS and their contribution to social information processing difficulties. Thus, investigating body-brain interaction in the context of social information processing could give a more elaborated understanding of the characteristics related to social functioning among individuals with ASD.

The anatomy and function of ANS have been studied for over 100 years (e.g., Langley, 1903). ANS is known to dynamically regulate the energy balance in body systems to adapt to environmental demands, that is, to maintain homeostasis, through the activation of the parasympathetic and sympathetic nervous systems (PSNS and SNS, respectively). While PSNS is predominant during conditions of rest promoting a state of recovery and energy conservation, activation of SNS prepares the organism to respond to challenging conditions and stressors resulting in a state of elevated activity or ‘fight or flight’ response. For historical reasons, these anatomically and functionally distinct branches of ANS are typically understood as a dichotomic system working in opposite directions. Although PSNS and SNS are indeed complementary in nature, their interaction is rather characterised by coactivity and reciprocity (Berntson & Cacioppo, 2004). The two branches of ANS and their dynamic interplay can be studied using recordings of pupil size, heart rate variability (HRV), skin conductance responses (SCR), and electrodermal activity (EDA) (Acharya et al., 2006; Berntson & Cacioppo, 2004; Mathôt, 2018; Posada-Quintero & Chon, 2020), that reflect the activation of PSNS and SNS to different degrees.

Although the role of ANS in homeostatic regulation is undisputable, its relevance for perception and processing of information is less established. A growing body of evidence suggests that ANS pathways interact with CNS even beyond homeostatic regulation, influencing affective and cognitive processes (Lacey & Lacey, 1978; Parviainen et al., 2022; Tsakiris & Critchley, 2016). For long, ANS activation has been considered to play a critical role in the experience of emotions (Damasio, 1998; Kreibig, 2010) and processing of emotional information (Quintana et al., 2012). During the past 10 years, the role of ANS in the perception and processing of non-emotional sensory information has been recognised and studied more comprehensively. A review by Critchley and Garfinkel (2018) addressed the manifold, yet tentative, interactions between physiology (e.g., cardiac, respiratory, and gastric activity) and cognitive processes (e.g., attention, perception, learning, and decision-making), and recent empirical studies have highlighted the coupling between the phase of bodily rhythms (e.g., cardiac cycle and respiration) and different perceptual and cognitive functions, such as oculomotor behaviour during visual sampling (Galvez-Pol et al., 2020) and encoding of information (Waselius et al., 2019). Since social functioning requires fluent processing of both emotional and non-emotional information, it is highly relevant to understand the role of ANS in processing of social information in more detail.

ANS pathways target several cortical areas (e.g., medial prefrontal cortex, PFC; anterior cingulate cortex, ACC; anterior insular cortex, AIC), some of which also seem to be relevant for processing of socially relevant information (Beissner et al., 2013; Frith & Frith, 2007; Van Overwalle, 2009). More specifically, the visceral target areas in the brain are shown to be linked with attaching value to objects, mentalizing, perspective taking and experience of emotions (Berntson & Khalsa, 2021; Frith & Frith, 2007; Van Overwalle, 2009). This gives a reason to consider the potential role of ANS-CNS interaction in processing of social information.

In typically developing individuals, emerging evidence suggests an interaction between neural and bodily rhythms. For instance, Herrero et al. (2018) have demonstrated links between the frequency of respiration and brain oscillatory activation, and Richter et al. (2017) have reported coupling between the phase of slow-oscillating gastric electric signalling and amplitude of the brain alpha oscillations. Moreover, Kluger and Gross (2020) have observed that the depth and phase of respiration modulate neural oscillatory activity. Besides the accumulating findings showing coupling between autonomic, enteric (governing gastrointestinal tract) and central nervous systems, the evidence from recent studies integrates the coupling between body and the brain with processing of external input (Kluger et al., 2021; Waselius et al., 2018), including processing of socially relevant information. For instance, Garfinkel et al. (2014) have shown that the phase of the cardiac cycle (i.e., diastole vs. systole) influences the neural processing of fear and threat. In addition, Candia-Rivera et al. (2022) have demonstrated that emotional stimuli modulate cardiac activity, which further plays a causal role in initiating a cortical response that is linked to emotions. Moreover, D’Hondt et al. (2010) have reported that early cortical responses to emotional pictures are significantly correlated with SCR. Eisenbarth et al. (2016), in turn, have found both common and measure-specific neural activation patterns predicting physiological responses to social threat. Aforementioned studies reflect the diversity of methodological approaches previously used to investigate body-brain interaction, and thus, an inclusive definition of body-brain interaction is emphasised in this systematic review as well.

It is generally assumed that deficits in social interaction and communication, the core features of ASD, may be due to atypical functional characteristics of the CNS. Indeed, accumulating body of evidence suggests that anatomical and functional neurobiological atypicalities are highly pervasive in ASD (Amaral et al., 2008; O’Reilly et al., 2017; Pardo & Eberhart, 2007; Stanfield et al., 2008; Wang et al., 2013). Recent meta-analyses have shown functional differences, such as hypoactivation, in brain regions relevant for social cognition (Di Martino et al., 2009; Patriquin et al., 2016) and in neural characteristics between individuals with ASD and typically developing individuals (Kang et al., 2018; Port et al., 2015). Especially, atypicalities regarding neural processing of social information, such as faces (e.g., Ammons et al., 2021; McPartland et al., 2004), emotions (e.g., Hall et al., 2003; Leung et al., 2018; Tseng et al., 2016), empathy (e.g., Fan et al., 2014; Schulte-Rüther et al., 2011) and joint attention (e.g., Redcay et al., 2013) have been observed among individuals with ASD. However, both similar brain activation between ASD and comparison groups as well as opposing findings have been reported, and thus, current evidence is considered ambiguous and inconsistent (e.g., Dufour et al., 2013; Hadjikhani et al., 2004; Pierce et al., 2004).

Interestingly, individuals with ASD have also been reported to demonstrate differential functional properties of ANS, such as chronic autonomic hypoarousal and hyperarousal (Bujnakova et al., 2016; Patriquin et al., 2019). According to a systematic review by Lydon et al. (2014), autonomic arousal during processing of socially relevant stimuli is also different among individuals with ASD when compared with typically developing individuals. For instance, lower amplitude of respiratory sinus arrhythmia (RSA), that is, the universally observed synchrony between respiration and heart rate, has been associated with increased experience of social stress (Cheng et al., 2020) and deficiencies in emotion recognition among individuals with ASD (Bal et al., 2010). Moreover, being an object of direct eye gaze has been shown to elicit differential patterns of arousal indexed by SCR in children with autism and in typically developing children (Kylliäinen & Hietanen, 2006). On the contrary, some studies have found no evidence of atypical autonomic responses to social stimuli in ASD (e.g., Louwerse et al., 2014). Generally, although some evidence exists, findings regarding ANS reactivity to social stimuli have been inconsistent and the relevance of ANS in social information processing remains unclear.

Taken together, there is extensive accumulating empirical evidence regarding neurobiological and functional atypicalities in the ANS and CNS among individuals with ASD. For approaching the relevance of these findings for behavioural features and experiences of individuals with ASD, it would be crucial to understand the association between ANS and CNS atypicalities. However, the studies focusing on both ANS and CNS functions in the same study, and especially their interaction, are more scattered. Indeed, research combining ANS and CNS measures in ASD does exist, but there is a lack of systematic examination of the accumulated evidence from the perspective of theoretically coherent rationale. Thus, there is a need for integrating the existing evidence of the interaction between ANS and CNS that may underlie social information processing difficulties among individuals with ASD.

Based on the existing theories and findings related to body-brain interaction in typically developing individuals, as well as literature regarding atypical neurophysiological reactivity and social information processing in individuals with ASD, we hypothesize that the characteristics of persons diagnosed with ASD may reflect altered ANS and CNS coupling during processing of sensory input, especially processing of socially relevant information. This assumption builds on the suggested role of ANS signalling in modulating initial perception of incoming stimuli and contributing to the cognitive top-down reappraisal of information flow also during social interaction (Badcock et al., 2017; Craig, 2014; Critchley & Harrison, 2013). Since ANS is evolutionarily older and automatically adjusts to internally or externally driven requirements (McEwen, 1998), our underlying assumption was that ANS activation modulates the processing of social information in the CNS. In other words, ANS could be regarded as a ‘filter’ that influences the interpretation of (external) sensory input in the CNS. Due to the observed atypicalities in ANS activation, this ‘filter’ is speculated to be deviant among individuals with ASD. If, by default, ANS reacts to sensory input with heightened arousal (i.e., hyperarousal) that stimulus may be interpreted as a threat leading to avoidance behaviour or aggression. On the other hand, if sensory input has only little or no effect on ANS activity (i.e., hypoarousal) that stimulus might not be regarded as salient resulting in underresponsiveness. In this systematic review we investigated whether the existing literature supports our speculation that the variation in psychophysiological state could explain both individual and moment-to-moment variation in processing and interpretation of social information.

Given the evidence for altered neural and physiological reactivity to social stimuli in ASD, our aim was to systematically review and qualitatively synthesise the evidence existing in support of or against our hypothesis of atypical (i.e., enhanced or decreased) body-brain interaction among individuals with ASD. Prior to the examination of ANS and CNS interactions, we first assembled the empirical evidence regarding coexisting differences in ANS and CNS activation during social information processing between individuals with ASD and typically developing individuals. Second, as our main question, we systematically reviewed the literature to investigate whether there is evidence that ANS-CNS interaction contributes to the processing of social information differently among individuals with ASD and typically developing individuals. To reach these aims we systematically reviewed studies that used simultaneous non-invasive measurement of ANS and CNS activity during social information processing among individuals with ASD and typically developing individuals.

## Methods

This systematic review was conducted according to PRISMA guidelines (Moher et al., 2009). However, this study or its protocol were not preregistered.

### Search Procedures

Comprehensive literature searches were conducted in five databases: PubMed, Medline, PsychInfo, PsychArticles, and Cinahl (until 12.1.2022). In all databases, search terms were inserted as free text into the search term field. Each search term consisted of a combination of keywords related to ASD, well-established non-invasive brain imaging methods and autonomic nervous system measures, such as (“autism” OR “autism spectrum disorder” OR “autistic” OR “Asperger”) AND (“MEG” OR “magnetoencephalography” OR “EEG” OR “encephalography” OR “fMRI” OR “functional magnetic resonance imaging”) AND (“SCR” OR “EDA” OR “skin conductance” OR “electrodermal” OR “galvanic skin response”).

**Table:** 

ASD	autism, autism spectrum disorder, autistic, Asperger
CNS	MEG, magnetoencephalography, EEG, electroencephalography, fMRI, functional magnetic resonance imaging
ANS	ECG, EKG, HRV, heart, heart rate, heart rate variability, cardiac; respiration, respiratory, breathing; pupil, pupillary response, blink, eye tracking, gaze; SCR, EDA, skin conductance, electrodermal, galvanic skin response; autonomic nervous system, parasympathetic, sympathetic, physiological response

### Study Selection

First, all the titles and abstracts of studies identified through the systematic search were screened for initial inclusion. If sufficient information was not provided in the title and abstract, full-text articles were retrieved. Studies were included for further assessment if they were peer-reviewed studies written in English. In addition, inclusion required that the ASD population was examined, study design included processing of socially relevant information, such as social cues (e.g., eye contact) and emotional expressions (e.g., facial expression of sadness) that are crucial for social understanding and social behaviour, and both ANS and brain activity were measured noninvasively. Since an inclusive definition of body-brain interaction was emphasised, causality between ANS and CNS measures was not required. The year of publication was not restricted.

Studies were excluded if either ANS or CNS outcome was missing or there was no social element (e.g., social cues, social interaction, emotions, faces, gaze direction, and empathy) in the study design. Furthermore, studies without ASD populations (e.g., siblings of individuals with ASD, individuals with increased likelihood of ASD, and individuals with subclinical ASD) were excluded. Case reports, systematic reviews, reviews, meta-analyses, study protocols, comments, opinions, and method papers were discarded. After removing duplicates, the reference lists for studies meeting the initial inclusion criteria were reviewed to identify additional studies for possible inclusion, but no new articles were found. Finally, the included full-text articles were assessed for eligibility. The following eligibility criteria (PICOTS) were used:

1. Population: individuals of all ages with a diagnosis of ASD
2. Intervention/exposure: socially relevant stimulus
3. Comparison: typically developing individuals
4. Outcomes: autonomic and central nervous system activity measured simultaneously
5. Time of publication: not restricted
6. Study type: peer-reviewed original research paper written in English

Studies that did not meet the eligibility criteria were excluded from the qualitative synthesis.

### Data Extraction

Studies meeting the eligibility criteria were summarised in terms of participant characteristics, experimental design, and outcome variables (i.e., ANS and CNS responses during social information processing). In addition, clinical information relevant for assessing the applicability and interpretation of the findings was extracted from the data. Thus, the following objective data was extracted from each study: gender and age of participants, the total number of participants in each group, criteria to match the ASD and comparison groups, information regarding ASD diagnosis (e.g., diagnostic criteria and methods), social stimuli used in the experiment, and measures of brain and ANS activity along with their outcomes. Where applicable, co-occurring conditions and the use of medication were also extracted to assess the homogeneity of comparison groups.

### Quality Assessment

Since no tools existed directly applicable for quality assessment of non-interventional, cross-sectional (neuro)physiological studies, we created a quality assessment checklist in accordance with recommendations for the assessment of methodological quality standards (Farrington, 2003) and the quality assessment measure devised by Lydon et al. (2014). Furthermore, criteria specific to research involving individuals with ASD were developed based on the reviews by Jarrold and Brock (2004) and Cohen et al. (2011).

The quality assessment checklist used to evaluate the studies included in this systematic review consists of 12 criteria that are separated into five categories: descriptive validity, internal validity, external validity, construct validity, and statistical conclusion validity (Farrington, 2003). Each criterion is scored 0 – 2 and defined either as high (2 = criterion is met), moderate (1 = criterion is partially met), or low (0 = criterion is not met) depending on the degree to which each criterion is met. Detailed description of the criteria and the assessment of the methodological quality of each study is represented in the Supplementary Material.

### Reliability of Search Procedures and Inter-Rater Agreement

To ensure the accuracy and reliability of the systematic literature search, authors 1 and 3 determined the detailed search terms, initial inclusion criteria and eligibility criteria. Then author 1 conducted the systematic literature searches and screened the articles for initial inclusion. Full-text review and assessment for eligibility were conducted independently by authors 1 and 3. Agreement on the eligibility of the articles was obtained on 28 of the 30 studies, that is, 93%. Disagreement occurred because the two studies were included by one author and excluded by the other. The agreement was achieved through consultation with author 2. Thereafter, author 1 extracted the relevant information from each eligible article. Similarly, the quality assessment of included studies was carried out by author 1. The accuracy of data extraction and appraisal of the studies was assessed by authors 2 and 3. If non-agreement emerged, consensus between authors was achieved through discussion and the data extracted and the appraisal of the study was revised accordingly.

## Results

### Studies Identified

**Fig. 1:**
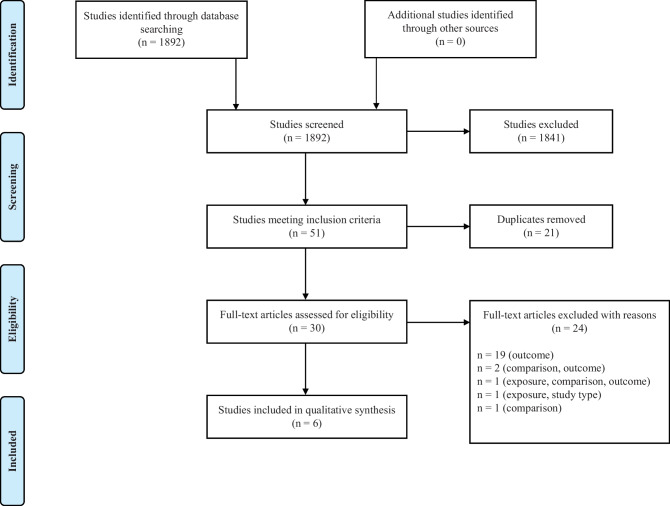
PRISMA flow diagram for systematic review literature search. Adapted from Moher et al. (2009)

Overall, the systematic literature searches identified 1892 studies (see Fig. 1). After screening the titles and abstracts for initial inclusion criteria, irrelevant studies were excluded (*n* = 1841) and duplicates were removed (*n* = 21). Thus, 30 full-text articles were further examined in detail to assess for eligibility. Twenty-four studies were excluded with the following reasons: missing an outcome measure or CNS and ANS activity were measured separately (*n* = 19); no comparison group as a comparison (*n* = 1); missing an outcome measure and no comparison group (*n* = 2); no social stimuli, missing an outcome measure and no comparison group (*n* = 1); and no social stimuli and ineligible study type (*n* = 1). Eventually, six studies fulfilled the eligibility criteria and were included in the qualitative synthesis.

### Sample Characteristics

Participant and study characteristics are summarised in Table 1. The year of publication ranged from 2005 to 2015. Included studies were conducted in Finland, Germany, the Netherlands, and the USA. The sample sizes ranged from 29 to 62. In total, the eligible studies included 248 participants, of which 128 were individuals with ASD and 120 were typically developing individuals. Furthermore, 97.2% of the participants were male and 2.8% were female. Two of the studies investigated children (Kylliäinen et al., 2012; Van Hecke et al., 2009), one study focused on teenagers (Dalton et al., 2005), and the remaining three studies involved adults (Althaus et al., 2015; Gu et al., 2015; Krach et al., 2015) with and without ASD.

**Table 1:** Summary of eligible studies included in the systematic review

Study	Participants (n; male)	Age (years; mean, SD)	Criteria for matching	ASD diagnostic method	ANS measure	CNS measure
Althaus et al. (2015)	ASD 32 (32) TD 30 (30)	ASD 22.69 ± 4.83 TD 22.60 ± 3.21	age sex GIT-2 IQ ≥ 80	DSM-IV ADOS / SRS-A / AQ	ECG maximum cardiac deceleration; IBI_MAX_	EEG late long-lasting parietal positivity parietal cortex (channels Pz, P3 and P7)
Dalton et al. (2005)	ASD I: 14 (14) II: 16 (16) TD I: 12 (12) II: 16 (16)	ASD I: 15.9 ± 4.71 II: 14.5 ± 4.60 TD I: 17.1 ± 2.78 II: 14.5 ± 4.56	I: age sex II: age sex	I: DSM-IV ADI-R / clinical interview WRIT / Stanford-Binet test for general IQ II: DSM-IV ADI-R / clinical interview WRIT for general IQ	ET fixations pupil diameter	fMRI amygdala FG mFG OC OFG
Gu et al. (2015)	ASD 17 (17) TD 17 (17)	ASD 26.2 ± 6.4 TD 26.8 ± 7.8	age sex socioeconomic status WAIS-III FSIQ > 80	DSM-IV-TR ADI-R ADOS-G	SCR	fMRI AIC EBA LPFC
Van Hecke et al. (2009)	ASD 19 (18) TD 14 (10)	ASD 9.95 ± 1.62 TD 9.93 ± 1.59	age sex	ADOS-G K-BIT IQ ≥ 75	ECG respiratory sinus arrhythmia	EEG spectral alpha band power temporal-parietal cortex (channels P2, P4 and PO4)
Krach et al. (2015)	ASD All: 16 (16) ET: 11 (11) TD All: 16 (16) ET: 11 (11)	ASD All: 21.50 ± 2.9 ET: 20.90 ± 1.8 TD All: 24.31 ± 2.9 ET: 24.27 ± 3.3	age sex WAIS-R verbal IQ	DSM-IV & ICD-10 ADI-R ADOS-G WISC-IV	ET pupil diameter	fMRI ACC AIC
Kylliäinen et al. (2012)	ASD 14 (13) TD 15 (14)	ASD 13.00 ± 1.17 TD 12.83 ± 1.17	age sex WISC-III verbal and performance IQ	ADI-R ADOS	SCR	EEG frontal asymmetry of spectral alpha band power frontal cortex (channels F3 and F4)

ANS, autonomic nervous system; *CNS*, central nervous system; *ASD*, autism spectrum disorder; *TD*, typically developing; *EEG*, electroencephalography; *fMRI*, functional magnetic resonance imaging; *ECG*, electrocardiography; *ET*, eye−tracking; *SCR*, skin conductance response; *DSM*, Diagnostic and Statistical Manual of Mental Disorders; *ICD*, International Classification of Diseases; *ADI−R*, Autism Diagnostic Interview−Revised; *ADOS*, Autism Diagnostic Observation Schedule; *ADOS−G*, Autism Diagnostic Observation Schedule−Generic; *SRS−A*, Social Responsiveness Scale for Adults; *AQ*, Autism Spectrum Quotient; *WAIS*, Wechsler Adult Intelligence Scale; *WISC*, Wechsler Intelligence Scale for Children; *WRIT*, Wide Range Intelligence test; *K−BIT*, Kaufman Brief Intelligence Test; *IQ*, Intelligence Quotient; *FSIQ*, Full Scale Intelligence Quotient; *FG*, fusiform gyrus; *mFG*, medial frontal gyrus; *OG*, occipital gyrus; *OFG*, orbitofrontal gyrus; *AIC*, anterior insular cortex; *EBA*, extrastriate body area; *LPFC*, lateral prefrontal cortex; *ACC*, anterior cingulate cortex

In all six studies, clinical ASD diagnoses were confirmed using Autism Diagnostic Interview-Revised or Autism Diagnostic Observation Schedule. Three studies reported that participants with ASD met the diagnostic criteria described in the Diagnostic and Statistical Manual of Mental Disorders (DSM-IV) (Althaus et al., 2015; Dalton et al., 2005; Gu et al., 2015), and one study reported meeting the criteria described both in DSM-IV and in International Classification of Diseases, Tenth Revision (Krach et al., 2015). The remaining two studies (Kylliäinen et al., 2012; Van Hecke et al., 2009) did not specify the criteria for the clinical ASD diagnosis. All of the participants with ASD had either Full Scale Intelligence Quotient (IQ) ≥ 75, Verbal IQ or Performance IQ within normal range. Cognitive assessment was conducted using Wechsler Adult Intelligence Scale (WAIS-III, WAIS-R), Wechsler Intelligence Scale for Children (WISC-III, WISC-IV), Groninger Intelligentie Test, Kaufman Brief Intelligence Test, Wide Range Intelligence test or Stanford-Binet test for general IQ. ASD and comparison groups were matched for age and sex in five studies. In addition, four studies matched the comparison group for intelligence (Althaus et al., 2015; Gu et al., 2015; Krach et al., 2015; Kylliäinen et al., 2012), and one study also considered the socioeconomic status (Gu et al., 2015).

Only one study reported that the participants were taking medication (Van Hecke et al., 2009). Two studies stated that the participants were free from the use of psychoactive and neuroleptic medication (Althaus et al., 2015; Gu et al., 2015). The use of medication was not reported in three studies (Dalton et al., 2005; Krach et al., 2015; Kylliäinen et al., 2012). In all six studies, comparison participants were healthy, typically developing individuals with no psychiatric or developmental diagnosis. Only one study reported using neurological and psychiatric conditions as exclusion criteria for individuals with ASD (Gu et al., 2015), otherwise, co-occurring conditions were not reported.

### Experimental Designs

As indicators of ANS activation, two studies used heart-rate related measures, that is, the maximum cardiac deceleration and RSA (Althaus et al., 2015; Van Hecke et al., 2009), and another two studies used eye-tracking measures, that is, pupil diameter and fixations (Dalton et al., 2005; Krach et al., 2015). Moreover, SCRs were measured in two studies (Gu et al., 2015; Kylliäinen et al., 2012). Since pupil diameter, fixation patterns and SCR are regarded as measures of sympathetic activity and maximum cardiac deceleration and RSA are considered measures of parasympathetic activity, most of the studies measured the sympathetic division of ANS (Dalton et al., 2005; Gu et al., 2015; Krach et al., 2015; Kylliäinen et al., 2012) and the remaining two studies measured the parasympathetic division (Althaus et al., 2015; Van Hecke et al., 2009). CNS activation was measured using electroencephalography in three studies (Althaus et al., 2015; Kylliäinen et al., 2012; Van Hecke et al., 2009) and functional magnetic resonance imaging in the remaining three studies (Dalton et al., 2005; Gu et al., 2015; Krach et al., 2015).

Social stimuli used in the experiments (see Table 2) included affective pictures from the International Affective Picture System (IAPS) database with and without humans (Althaus et al., 2015), images of emotional and neutral faces (Dalton et al., 2005), images of familiar and unfamiliar faces (Dalton et al., 2005; Kylliäinen et al., 2012), videos of familiar and unfamiliar people (Van Hecke et al., 2009), as well as images depicting socially (Krach et al., 2015) and physically painful and non-painful situations (Gu et al., 2015; Krach et al., 2015). Furthermore, Gu et al. (2015) and Krach et al. (2015) used partly similar pictures as physically painful stimuli in their experiments. Most of the studies were conducted using static images, but one study used videos (Van Hecke et al., 2009) and another study used static pictures that were ‘looming’ towards the participant (Kylliäinen et al., 2012).

**Table 2:** Summary of social stimuli used in the experiments

Study	Process	Social stimuli	Experimental design
Althaus et al. (2015)	orienting to social information	affective images International affective picture system (IAPS)	randomised double-blind placebo-controlled crossover trial neutral / positive / negative, humans / scenes
Dalton et al. (2005)	I: emotion discrimination II: facial recognition	I: images of emotional and neutral faces Karolinska directed emotional faces (KDEF) II: images of familiar and unfamiliar faces	I: deciding whether the image is emotional or neutral emotional / neutral, looking straight / quarter-turned with eyes averted II: deciding whether the image was familiar or unfamiliar familiar / unfamiliar, human / object
Gu et al. (2015)	empathy	images of painful and nonpainful situations	judging whether the person in the image is suffering from pain or not first-person perspective left / right, hands / feet, painful / nonpainful
Van Hecke et al. (2009)	social engagement	videos of familiar and unfamiliar people reading stories from The Time Warp Trio books	videos of a familiar person reading a story, an unfamiliar person reading a story and objects moving to classical music
Krach et al. (2015)	empathy	PP: images of vicarious physical pain SP: images of vicarious social pain	PP: estimating the intensity of physical pain the depicted person would experience first-person perspective left / right, hands / feet, painful / nonpainful SP: evaluating the intensity of vicarious social embarrassment hand-drawn sketches of a person in socially undesirable and neutral public scenarios
Kylliäinen et al. (2012)	approach – avoidance	images of familiar and unfamiliar faces	images of faces looming towards the participant eyes shut / eyes open / eyes wide open frontal views of cars looming towards the participant

PP, physical pain; *SP*, social pain

Only one study reported measuring baseline activity (i.e., resting state measurement in the absence of any task or stimulation) separately from stimulus presentation (Van Hecke et al., 2009), otherwise baseline-adjusted ANS and CNS measures were used for data analysis, except in the study by Dalton et al. (2005) in which no baseline-adjustment was used. Three of the studies used non-social stimuli as a control condition; pictures with scenes (Althaus et al., 2015), videos of objects (Van Hecke et al., 2009), and images of cars (Kylliäinen et al., 2012). Moreover, Dalton et al. (2005) used images of familiar and unfamiliar objects as a control condition in facial recognition task, but the results were not discussed in the article. Additionally, the use of a control condition was not reported in the emotion discrimination task (Dalton et al., 2005). Furthermore, in two studies, the use of a non-social control condition was not reported (Gu et al., 2015; Krach et al., 2015). No differences in ANS or CNS activation were found between individuals with ASD and typically developing individuals in the non-social control conditions (Althaus et al., 2015; Kylliäinen et al., 2012; Van Hecke et al., 2009).

### Results of Individual Studies

#### ANS activation during social information processing

The main findings of each study are summarised in Table 3. When examining the ANS responses to social stimuli, three of the studies reported higher levels of arousal, that is, hyperarousal in individuals with ASD compared with the typically developing individuals (Dalton et al., 2005; Gu et al., 2015; Van Hecke et al., 2009). One study, in turn, reported lower levels of arousal, that is, hypoarousal in the ASD group (Kylliäinen et al., 2012) and another found no significant differences between the ASD and typically developing (TD) groups in ANS reactivity to social stimuli (Althaus et al., 2015). Furthermore, the study by Krach et al. (2015) found hypoarousal in social pain task and no group differences in physical pain task. Hyperarousal was reflected as lower RSA to videos of unfamiliar people (Van Hecke et al., 2009), atypical fixation patterns to images of faces (Dalton et al., 2005) and enhanced SCR to physically painful images (Gu et al., 2015). Hypoarousal, in turn, was reflected as smaller pupil diameter to images depicting social pain (Krach et al., 2015) and attenuated SCR to images of faces (Kylliäinen et al., 2012).

**Table 3:** Summary of main findings. Comparison of ANS and CNS measures between ASD and TD groups along with possible ANS – CNS interaction during social information processing are reported

Study	ANS	CNS	ANS-CNS interaction (ASD)	ANS-CNS interaction (TD)
Althaus et al. (2015)	no difference	no difference	N/A	N/A
Dalton et al. (2005)	I: hyperactivation II: hyperactivation	I: hyperactivation (amygdala & OFG) hypoactivation (FG, OG & mFG) II: hyperactivation (amygdala) hypoactivation (FG & OG) no difference (OFG & mFG)	I: positive association II: positive association	I: no association II: no association
Gu et al. (2015)	hyperactivation	hyperactivation (AIC & EBA) hypoactivation (LPFC)	positive association	positive association
Van Hecke et al. (2009)	hyperactivation	no difference	no association	no association
Krach et al. (2015)	PP: no difference SP: hypoactivation	PP: no difference SP: hypoactivation	PP: positive association SP: no association	PP: positive association SP: positive association
Kylliäinen et al. (2012)	hypoactivation	hypoactivation	N/A	N/A

ANS, autonomic nervous system; *CNS*, central nervous system; *ASD*, autism spectrum disorder; *TD*, typically developing; *N/A*, not reported; *OFG*, orbitofrontal gyrus; *FG*, fusiform gyrus; *OG*, occipital gyrus; *mFG*, medial frontal gyrus; *AIC*, anterior insular cortex; *EBA*, extrastriate body area; *LPFC*, lateral prefrontal cortex; *PP*, physical pain; *SP*, social pain

#### CNS activation during social information processing

Differences in CNS responses to social stimuli between individuals with ASD and typically developing individuals were found in four studies, whereas two studies reported no group differences (Althaus et al., 2015; Van Hecke et al., 2009). Krach et al. (2015) reported no group differences in hemodynamic brain responses when images depicting vicarious physical pain were presented, but they also reported less pronounced brain activation overall and specific decreases in AIC and ACC to images depicting vicarious social pain among individuals with ASD. Kylliäinen et al. (2012) reported no approach-related, that is, relative left-sided frontal electrophysiological brain activation to images of faces in the ASD group that was observed in the typically developing individuals. The remaining two studies found both specific increases and decreases in CNS activation to social stimuli. Dalton et al. (2005) showed greater hemodynamic activation in amygdala and orbitofrontal gyrus along with less activation in the fusiform gyrus (FG), occipital gyrus and middle frontal gyrus during emotion discrimination among individuals with ASD. Greater activation was found in the amygdala and less activation in FG and occipital cortex during facial recognition. Gu et al. (2015) showed greater hemodynamic activation in AIC and extrastriate body area along with decreased activation in lateral PFC to physically painful images in the ASD group. Although Gu et al. (2015) and Krach et al. (2015) used similar pictures depicting physical pain, their findings were contradictory. Furthermore, opposing findings regarding AIC activation, that is decreased AIC activation during vicarious social pain task and increased AIC activation during physical pain task, were reported (Gu et al., 2015; Krach et al., 2015). Considering the experimental settings, these opposing findings could be explained by the differences in the task demands as participants were either judging whether the individual in the image was suffering from pain or not (Gu et al., 2015) or estimating the intensity of physical and social pain depicted in the image (Krach et al., 2015).

#### Interaction between ANS and CNS during social information processing

The association between ANS and CNS measures was not directly investigated in two studies (Althaus et al., 2015; Kylliäinen et al., 2012). Van Hecke et al. (2009) reported that RSA and temporal-parietal brain activation were not associated in either of the groups when videos of familiar and unfamiliar people were presented. Similarly, Krach et al. (2015) found no association between pupil dilation and hemodynamic responses in AIC and anterior cingulate gyrus (ACG) in the ASD group when images depicting vicarious social pain were represented, although the variability in the pupil dilation covaried with the dynamics in hemodynamic responses in the comparison group. Furthermore, both ASD and TD groups showed similar covariation between pupil diameter and hemodynamic responses to images depicting vicarious physical pain (Krach et al., 2015). Dalton et al. (2005) and Gu et al. (2015), in turn, reported positive associations between ANS and CNS measures. Dalton et al. (2005) showed that activation in the amygdala and FG were strongly and positively associated with the amount of time spent fixating on the eyes in the ASD group, but not in the comparison group. Furthermore, Gu et al. (2015) reported an association between SCR and activity in the AIC in both groups. It is worth noting that the correlation between these measures was enhanced in the ASD group in comparison with the comparison group.

Although using partly similar pictures depicting physical pain, Gu et al. (2015) reported an enhanced association between ANS and CNS among individuals with ASD, whereas Krach et al. (2015) reported that the interaction between ANS and CNS was similar in both groups. Moreover, Krach et al. (2015) found no association between ANS and CNS during vicarious social pain task among individuals with ASD. These somewhat contradictory findings may be explained by the differences in task demands, as mentioned earlier, because judging whether someone is suffering pain or not and estimating the intensity of pain (physical pain or social embarrassment) may involve critically different processes and neural networks. Moreover, the vicarious physical and social pain tasks used in the study by Krach et al. (2015) required taking another person’s perspective. Thus, also the level of cognitive and bodily engagement may have differed between these tasks resulting in the observed differences. Furthermore, the brain regions of interest (excl., AIC) differed between these studies, which may also explain these opposing findings.

#### Associations between ANS or CNS activation and participant characteristics

Associations between ANS or CNS activation and participant characteristics were investigated in three studies. Dalton et al. (2005) reported a marginal correlation between neural activation and IQ for the ASD group, otherwise no significant correlations were found. Krach et al. (2015), reported that in the ASD group activation within the AIC during social pain task, but not during physical pain task, was inversely related to autism symptom severity in the domain of social affect (ADOS). Van Hecke et al (2009) found no significant correlations between IQ and physiological data, whereas higher baseline RSA correlated with higher levels of social skills and lower levels of problem behaviours on the Social Skills Rating System (SSRS), as well as lower (i.e., more typical) social communication scores, autistic mannerism scores, and total scores on the Social Responsiveness Scale (SRS) when the whole sample was investigated. Moreover, higher baseline alpha activity measured with EEG was related to lower (i.e., more typical) social motivation scores. When groups were tested separately, no significant correlations were found in the group of typically developing individuals. In the ASD group, higher baseline RSA was correlated with higher levels of social skills on the SSRS, and higher baseline alpha activation was related to lower (i.e., more typical) social motivation scores. These results imply that the activation of ANS or CNS may, at least partly, reflect the heterogeneity of individual characteristics observed among individuals with ASD.

### Methodological Quality

**Fig. 2:**
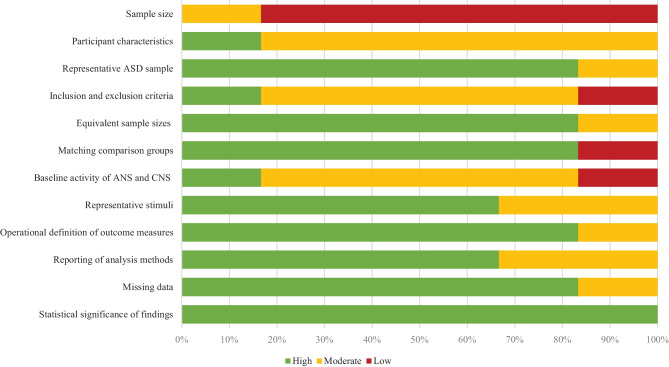
Summary of the assessment of methodological quality. The quality of each criterion (high, moderate, low) is presented as percentages across all included studies. Abbreviations: ANS, autonomic nervous system; CNS, central nervous system; ASD, autism spectrum disorder

The summary of the assessment of methodological quality is presented in Fig. 2. Overall, the methodological quality of the eligible studies included in this systematic review was adequate (from moderate to high). Limitations mainly concerned the descriptive validity of the studies, such as small sample sizes (6/6 studies), insufficient description of participant characteristics (5/6 studies) as well as inclusion and exclusion criteria (5/6 studies). Moreover, the internal validity in most of these studies was compromised due to the lack of baseline measurements of ANS and CNS activity (5/6 studies). In turn, statistical conclusion validity, construct validity and external validity were high across all the studies. For instance, operational definitions of the outcome measures (5/6 studies), representativeness of the stimuli (4/6 studies), appropriate reporting of analysis methods (4/6 studies) and missing data (5/6 studies) as well as the investigation of statistical significance (6/6 studies) were mainly of high quality.

## Discussion

### Summary of the Findings

The aim of this article was to systematically review and qualitatively synthesize the empirical evidence regarding differences in ANS and CNS activation as well as body-brain interaction during social information processing between individuals with ASD and typically developing individuals. The focus was on studies where both ANS and CNS activity were simultaneously measured. First, differences in ANS and CNS activations between individuals with ASD and typically developing individuals were examined separately. As expected, based on the previous literature (e.g., Lydon et al., 2014), both ANS hypoarousal (attenuated SCR, smaller pupil diameter) and hyperarousal (enhanced SCR, lower RSA, atypical eye fixation pattern) were observed during processing of social information among individuals with ASD. Moreover, two of the studies found no differences in autonomic arousal between typically developing individuals and individuals with ASD (Althaus et al., 2015; Krach et al., 2015 [physical pain]).

Atypicalities in the CNS activation during social information processing were also reported among individuals with ASD. Differences were found in the hemodynamic activation of amygdala, AIC, and ACC (Dalton et al., 2005; Gu et al., 2015; Krach et al., 2015 [social pain]), which are considered relevant in processing of social information (Van Overwalle, 2009). Moreover, Kylliäinen et al. (2012) demonstrated no approach-related electrophysiological brain activation that was observed among the typically developing individuals. However, there were also studies indicating similar brain activation in ASD and TD groups during social information processing (Althaus et al., 2015; Krach et al., 2015 [physical pain]; Van Hecke et al., 2009). Taken together, these observations provide initial evidence regarding both ANS and CNS atypicalities during processing of socially relevant information among some individuals with ASD, although the results are still inconsistent and context dependent.

As our main question we investigated whether the interaction between ANS and CNS is meaningful in understanding ASD, in other words whether ANS-CNS interaction contributes to the processing of social information differently among individuals with ASD and typically developing individuals. The interaction between ANS and CNS activation was not directly approached in two studies (Althaus et al., 2015; Kylliäinen et al., 2012) and one study (Van Hecke et al., 2009) reported no association between cardiac and electric brain activation patterns in either of the groups. In the remaining three studies, atypicalities in interaction between ANS and CNS were observed among individuals with ASD. The association between ANS and CNS during emotion discrimination and facial recognition was observed only among individuals with ASD, and not among typically developing individuals (Dalton et al., 2005). Based on these findings, Dalton et al. (2005) claim that atypical eye fixation pattern is associated with overarousal and further, the face-processing deficits in ASD result from hyperactivation in the central circuitry of emotion leading to heightened sensitivity to social stimuli.

When investigating empathy for physical pain, enhanced interaction between ANS and CNS activation in individuals with ASD were demonstrated (Gu et al., 2015). Thus, it was proposed that the heightened autonomic and cortical arousal might result in empathy deficits in ASD (Gu et al., 2015). Findings by Krach et al. (2015), in turn, suggested that the embodied representation of complex emotions such as empathy for social pain is reduced among individuals with ASD in comparison with the typically developing individuals, but basic abilities related to sharing another’s affect remain intact. Based on these findings, it can be speculated that among individuals with ASD the contribution of ANS on neural processing of empathy for physical pain is similar or even enhanced in comparison with typically developing individuals. However, there is no interaction between ANS and CNS during processing of vicarious social pain among individuals with ASD. Although the small number of studies does not allow to make strong conclusions, the integration of the above findings would allude that there indeed seems to be an altered contribution of ANS-CNS interaction among individuals with ASD during processing of socially relevant information, but more evidence is needed to understand this body-brain interaction, and especially the causal relations, in social contexts more accurately.

Altogether, our ultimate aim was to synthesize the evidence existing in support of or against our hypothesis of atypical body-brain interaction among individuals with ASD during social information processing. Based on the fairly limited evidence, interaction between the ANS and CNS appears to be enhanced in ASD (Dalton et al., 2005; Gu et al., 2015). This was demonstrated between fixation patterns and hemodynamic responses, as well as SCR and hemodynamic responses during processing of images of faces and physically painful situations, respectively. On the contrary, ASD was characterized with diminished interaction between ANS and CNS in response to socially painful situations (Krach et al., 2015). Thus, tentative empirical evidence exists in support of our hypothesis of atypical (i.e., enhanced or decreased) body-brain interaction among individuals with ASD during processing of socially relevant information.

On the contrary, no association between RSA and spectral alpha band power in either of the groups was found when videos of familiar and unfamiliar people were presented, thus not supporting our assumption but not rejecting it either (Van Hecke et al., 2009). However, this contradictory finding may be explained by factors related to the experimental setting. Since videos were used as social stimuli (Van Hecke et al., 2009), whereas other studies used static images, the complexity and ecological validity of the videos (incl., listening to a story, familiar and unfamiliar readers, naturalistic gestures, direct eye gaze) may have affected the findings. Additionally, watching a video may be more activating overall leading to differential activation patterns in comparison with static images. Furthermore, Van Hecke et al. (2009) only investigated right temporal-parietal activity, whereas the possible associations between ANS and CNS could have been localized to other brain areas (cf., Beissner et al., 2013). Therefore, it is possible that the CNS measure used was not optimal to investigate body-brain interaction. Taken together, the systematic review of the literature mainly supports our speculation of atypical body-brain interaction among individuals with ASD, but there is also evidence against our speculation.

### Limitations and Recommendations for Future Research

The results of this systematic review must be considered in the context of several limitations. First, the extensive literature search identified over 1800 studies but only six of them met the eligibility criteria, indicating that there is plenty of research regarding ANS and CNS activation, but only a few studies have investigated ANS and CNS activation simultaneously during social information processing. Thus, our literature search was rather inclusive and may therefore be criticized for the lack of specificity. However, our aim was to ensure that as many relevant studies as possible were included in the systematic review and hence the sensitivity of the literature search was emphasised. It must also be noted that due to the restrictions regarding the small number of included studies and the heterogeneity of the findings, conducting a meta-analysis was not reasonable.

One of the main limitations in this systematic review was that the sample sizes in each study were relatively small. Moreover, most of the studies only involved individuals with ASD that had either FSIQ ≥ 75 or Verbal or Performance IQ within the normal range. Since majority (97.2%) of the participants in the included studies were male, these results may not be applicable for females or non-binary people. Thus, there is a risk of bias in this systematic review and the results cannot be generalised to the entire autism spectrum as such. In the future, studies with larger and more representative samples are needed.

In addition, there was high variability in the results across the studies included in this systematic review that may be explained by several factors. First, different combinations of ANS and CNS measures (e.g., RSA – EEG and pupillometry – fMRI) were used in these studies. Consequently, since SCR, pupillometry and fixation patterns mainly reflect sympathetic activity and maximum cardiac deceleration and RSA are considered mainly parasympathetic, some studies of the present review measured the sympathetic division of ANS (i.e., SNS), whereas others focused on the parasympathetic branch (i.e., PSNS). Furthermore, there were differences across studies in the neural features investigated (e.g., hemodynamic responses or electric activation with focus on time- or frequency-domain characteristics and differences in the brain regions of interest). Moreover, some of the interpretations given to the selected ANS and CNS measures are highly context dependent or not well-established. For instance, while most of the studies used direct measures of ANS activation, sometimes also indirect derivative measures, such as fixations patterns, were used (Dalton et al., 2005). Typically, eye-tracking is used to ensure similar viewing patterns between comparison groups and to control for the influence of viewing pattern on brain activation (e.g., Greene et al., 2011; von dem Hagen et al., 2014). Dalton et al. (2005) regarded atypical eye fixations as an indirect measure of ANS activation reflecting overarousal mediated by activation of limbic areas and not just differential pattern of scanning faces. Furthermore, some of the used brain imaging measures, such as frontal asymmetry (Kylliäinen et al., 2012) have been considered inconsistent and, to some extent, controversial (e.g., Smith et al., 2017).

Second, a wide variety of tasks and social stimuli were used, so the variation in task demands, cognitive effort and emotional aspects of the experimental designs may explain the variability in the results. Furthermore, tasks and stimuli also varied in the degree to which they can capture the complexity and authenticity of social information processing in the real world. Taking authenticity into consideration is highly important, because previous studies have found differences in autonomic and neural responses to socially relevant stimuli when comparing static pictures, videos, and live interaction (Hietanen et al., 2020; Pönkänen et al., 2011). It is also worth mentioning that none of the included studies used natural social communication or interaction as a stimulus or task. These kinds of natural experimental settings are shown to be feasible (e.g., Karvonen et al., 2016; Silvennoinen et al., 2022; Stevanovic et al., 2019) and would be essential in demonstrating whether ANS-CNS interaction influences the day-to-day social functioning among individuals with ASD. Although the high variability in experimental settings is beneficial for developing novel methodological approaches, standardised way of conducting studies is required for replicating previous findings and increasing our understanding of ASD.

Third, it is possible that the variability in the results reflects the heterogeneity of individual characteristics across the autism spectrum. Even though criteria for matching (e.g., age, gender, IQ) were well reported in each study, the exclusion criteria were vaguely defined. For instance, the use of medication was not systematically reported. Only one study (Gu et al., 2015) reported using neurological and psychiatric conditions as exclusion criteria for participants with ASD, but otherwise co-occurring conditions were not explicitly reported. Thus, assessing the possible confounding factors such as co-occurring developmental (e.g., attention-deficit/hyperactivity disorder, sensory processing disorder, specific language impairment), mental health (e.g., depression, anxiety, obsessive–compulsive disorder) and physical conditions (e.g., cardiovascular disease) or personality traits (e.g., alexithymia), was not possible. Since these background factors may also add complexity to understanding the body-brain interaction across autism spectrum, they should be either excluded in advance or eventually controlled for using statistical analysis approaches (Jarrold & Brock, 2004; Lydon et al., 2014).

Due to the high heterogeneity of ASD, it is important that participant characteristics are described also beyond the diagnosis (e.g., detailed description of social functioning, communication skills and sensory processing), enabling more accurate comparison of results between studies and evaluation of generalisability of the results across the autism spectrum. Alternatively, recruiting a group of individuals with a specific trait related to social functioning would allow investigating how body-brain interaction is associated with certain social information processing features among individuals with ASD. Furthermore, to take neurodiversity into account, recruiting both well-matched typically developing individuals and several comparison groups, such as individuals with other developmental or neuropsychiatric conditions, would be beneficial in establishing the variance of the autonomic and neurophysiological responses during social information processing. Taken together, due to the small number of eligible studies included in this systematic review, as well as high variability in physiological and neural measures, social stimuli and sample characteristics, the conclusions of this systematic review need to be interpreted with caution.

Despite rapidly accumulating empirical evidence, the knowledge and understanding of the underlying neural and physiological mechanisms associated with ASD have not improved at the same pace due to the lack of replicability. Notably, the variability across studies in applied measures, task demands and stimuli, was observed in this systematic review as well. Hence, a clear shortcoming in the reviewed studies, from the perspective of our research question, is the lack of systematic protocol for using simultaneous ANS and CNS recordings. It is worth noting that although the synthesis of the results suggests atypical body-brain interaction among individuals with ASD, the involved studies were originally designed for different purposes. Thus, the methodological choices may not have been optimal for investigating the interaction between ANS and CNS during social information processing. To examine body-brain interaction in a systematic manner and to improve our understanding of social information processing among individuals with ASD in the future, a consistent standardised way of conducting and reporting (neuro)physiological research is needed. Here we suggest key notions that would help to harmonize the experimental settings when examining the interaction between ANS and CNS activation during processing of socially relevant information among individuals with ASD:

- The *use of existing standardised guidelines for conducting and reporting neurophysiological research* (e.g., Camm et al., 1996; Gross et al., 2013) would enable quality control and reproducibility of studies. The lack of standardised practices in conducting and reporting neurophysiological research involving individuals with ASD has been pointed out in several reviews (Lydon et al., 2014; Patriquin et al., 2019), also including our systematic review.
- *Correct selection, measurement, and interpretation of specific ANS recordings* along with detailed reporting of used measures and analysis pipelines is needed to improve the accuracy and replicability of the results. The quality and specific features of the raw signal (e.g., length of recording, sampling rate, and number of ectopic heartbeats) influence the validity and accuracy of ANS outcome measure computation and interpretation.
- When combining ANS and CNS measures, *methodological restrictions specific to the brain imaging modality* need to be acknowledged. For instance, physiological recordings can be effortlessly combined with EEG and MEG, while with fMRI they need specific technical considerations to ensure safety and quality of the data (Babiloni et al., 2009; Bulte & Wartolowska, 2017; Gray et al., 2009; Iacovella & Hasson, 2011).
- *Establishing the baseline variance* is necessary to determine whether the atypical characteristics observed in ASD reflect trait-like differences (i.e., differences in resting ANS or CNS activity) instead of state-like differences (i.e., differences in reactivity to stimuli or task induced conditions). Additionally, a non-social control condition would allow to assess specificity of possible differences in social tasks.
- *The use of standardised stimulus (e.g., pictures, videos, and other pre-prepared stimuli) sets or well-controlled authentic social settings* is recommended to increase the cross-study interpretation (see e.g., IAPS database; Lang et al., 2008). When applying experimental settings involving natural social interaction, detailed description of methods and experimental setting is important. The complexity and authenticity of the stimuli and tasks need to be considered when reporting and interpreting the results.
- *Acknowledging individual variability* and taking into account individual differences as well as intervening factors across the autism spectrum, instead of considering individuals with ASD as one group, facilitates cross-study comparison and increases the generalisability of the results.

To establish understanding on the role of ANS driven alterations in ASD, future studies that examine the relevance of body-brain interaction during social information processing would benefit from more strongly building on existing theories on the role of ANS in human experience and ANS-CNS interaction (e.g., Critchley & Harrison, 2013). It would also be essential to conduct experiments that directly test whether and how ANS activation modulates the processing of social information in the CNS among individuals with ASD and typically developing individuals. It is particularly challenging to go beyond correlative evidence to establish causal relations. For this, one approach is to modify the state of ANS, for example using relaxation techniques, medication, or even direct stimulation of the vagus nerve, and assess the modulatory effects of ANS both on the neural and behavioural level (Khalsa et al., 2018). Alternatively, experimental designs in which stimuli are presented during different phases of ANS rhythms (e.g., inspiration vs. expiration, cardiac systole vs. diastole) could be used in investigating body-brain-cognition coupling (Parviainen et al., 2022).

If our primary hypothesis on body-brain interaction is correct and ANS activation indeed modulates CNS activity and further the perception and interpretation of social information, testing and developing interventions that affect ANS activity (e.g., breathing and relaxation techniques, sensory stimulation, and animal-assisted therapy) could be beneficial. Especially, understanding how ANS reactions are linked with processing and interpretation of socially relevant information could increase self-knowledge and self-esteem of individuals with difficulties in social interaction. Moreover, improving self-regulation by learning to regulate ANS activation could support social functioning as well as wellbeing and participation of individuals with ASD. In the future, studies examining how body-brain interaction during development gives rise to characteristics associated with autism are needed. Furthermore, it would be of great importance to understand how genetic, environmental, and individual factors (e.g., temperament, interoceptive awareness and sensitivity, sensory processing atypicalities) influence the manifestation of body-brain interaction throughout the lifespan.

From the methodological point of view, a greater focus on combining two or more measures of ANS activation would also improve our understanding regarding the interplay between PSNS, SNS and CNS during processing of socially relevant information. Indeed, each recording (e.g., EDA, HRV, pupillometry) alone captures a limited view on the ANS functions typically emphasising either sympathetic or parasympathetic division. In general, acknowledging the rich and complementary information available in different ANS recordings is likely to increase our understanding of the role of bodily states for experience. Moreover, future methodological advancements in mobile EEG, MEG, and functional near-infrared spectroscopy as well as in hyperscanning enables to test body-brain interaction also in ecologically valid experimental settings, such as during natural social interaction. The studies conducted in natural settings should, however, be accompanied by studies, where the parameters can be strictly controlled and manipulated to achieve reliable interpretation for the different features reflecting ANS-CNS coupling.

In the future, research assessing the influence of individual factors, such as gender, co-occurring conditions, and personality traits, could clarify the individual variability in body-brain interaction. Since there is a strong gender bias towards clinical presentation of ASD in males (Loomes et al., 2017), future studies should take gender bias as well as gender diversity into account. Furthermore, the use of several comparison groups would enable studying possible transdiagnostic mechanisms and processes as well as diagnosis-specific phenomena.

## Conclusions

In summary, the results of this systematic review demonstrate coexisting but context dependent ANS and CNS atypicalities during processing of socially relevant information among individuals with ASD. Furthermore, there is indication of altered reactivity and/or trait features in ANS activity among individuals with ASD that may contribute to social information processing by influencing the perception and processing of socially relevant stimuli in the brain. However, more empirical evidence is needed to establish our knowledge of the body-brain interaction and its role in social functioning among individuals with ASD. Notably, standardised research practices and rigorous use of methods should be fostered to achieve reliable increment of knowledge regarding the underlying neural and physiological mechanisms contributing to alterations in social functioning and social information processing. Furthermore, understanding how individuals with ASD process socially relevant information is crucial for development of support services and interventions that aim at improving the well-being and participation of individuals with ASD.

### Supplementary Information

Below is the link to the electronic supplementary material.

Supplementary file1 (DOCX 37 KB)

Supplementary file2 (DOCX 35 KB)

## Data Availability

All the studies included in this systematic review are publicly available.
