# Supplementary material for: Coactivation of Autonomic and Central Nervous Systems During Processing of Socially Relevant Information in Autism Spectrum Disorder: A Systematic Review
Source: Neuropsychol Rev. 2023 Feb 27;34(1):214–31. doi: 10.1007/s11065-023-09579-2 (PMC10920494; doi:10.1007/s11065-023-09579-2)
Supplement: Supplementary file 1 — Supplementary file1 (DOCX 37 KB) [file 11065_2023_9579_MOESM1_ESM.docx]

**Neuropsychology Review**

**Coactivation of autonomic and central nervous systems during processing of socially relevant information in autism spectrum disorder: a systematic review**

Suvi Karjalainen *, Tuija Aro, Tiina Parviainen

* Corresponding author

Department of Psychology & Centre for Interdisciplinary Brain Research,

University of Jyväskylä, Jyväskylä, Finland

E-mail: suvi.k.karjalainen@jyu.fi

**Supplementary material**

**SM1** Criteria used to assess the quality of the eligible studies included in the systematic review

|  | **Criteria** | **High = 2** | **Moderate = 1** | **Low = 0** |
| --- | --- | --- | --- | --- |
| **Descriptive validity** | Sample size | > 30 participants per group or condition | 20 – 30 participants per group or condition | < 20 participants per group or condition |
|  | Participant characteristics | Following characteristics are outlined: age, gender, intellectual functioning, medication use, co-morbid psychiatric or neurodevelopmental diagnoses | Only 3-4 of the mentioned characteristics are reported | Less than 3 of the mentioned characteristics are reported |
|  | Representative ASD sample | ADI or ADOS or a clinical interview is used to confirm the ASD diagnosis | Only a questionnaire is used to confirm the ASD diagnosis | No sufficient assessment is conducted or reported |
|  | Inclusion and exclusion criteria | Inclusion and exclusion criteria are stated and similar for comparison groups (excluding ASD diagnosis) | Inclusion and exclusion criteria are stated, but they are not similar for comparison groups | Inclusion and exclusion criteria are not reported |
| **Internal validity** | Equivalent sample sizes in comparison groups | Groups are of equal sizes (i.e. size is more than 80% of the size of the comparison group) | Groups are not of equal sizes (i.e. size is 50-80% of the size of the comparison group) | Groups are not of equal sizes (i.e. size is less than 50% of the size of the comparison group) |
|  | Attempt of matching the ASD and control group | Comparison groups are matched on two or more factors (e.g. age, gender) | Comparison groups are matched on one factor | Comparison groups are not matched |
|  | Baseline activity of ANS and CNS is considered in analyses | Separate baseline activity of ANS and CNS is measured and considered in the analyses | Baseline-correction is considered in the analyses, but no separate baseline activity is measured | Baseline activity is not measured or considered in the analyses |
| **External validity** | Stimuli are representative to those encountered in everyday life | Stimuli that everyone may counter in their everyday life | Stimuli that some people may encounter in their everyday life | Stimuli that are not encountered in everyday life |
| **Construct validity** | Operational definition of outcome measures | ANS and CNS outcomes are operationally defined | ANS or CNS outcome is operationally defined | ANS and CNS outcomes are not operationally defined |
| **Statistical conclusion validity** | Appropriate reporting of analysis methods | All conducted analyses are reported in detail | Conducted analyses are partly reported | Analysis methods are not reported |
|  | Missing data | Exclusion of participants and the reason for exclusion is described OR It is stated that data was available from all participants | Exclusion of participants or the reason for exclusion is not described. | Data availability, exclusion and missing data are not reported |
|  | Statistical significance of findings is investigated | Yes | Partially | No |

*^Abbreviations: ANS, autonomic nervous system; CNS, central nervous system; ASD, autism spectrum disorder; ADI, Autism Diagnostic Interview; ADOS, Autism Diagnostic Observation Schedule^*

**SM2** Summary of the assessment of methodological quality

|  | **Descriptive validity** | | | | **Internal validity** | | | **External validity** | **Construct validity** | **Statistical conclusion validity** | | |
| --- | --- | --- | --- | --- | --- | --- | --- | --- | --- | --- | --- | --- |
|  | **Sample size** | **Participant characteristics** | **Representative ASD sample** | **Inclusion and exclusion criteria** | **Equivalent sample sizes** | **Matching comparison groups** | **Baseline activity of ANS and CNS** | **Representative stimuli** | **Operational definition of outcome measures** | **Reporting of analysis methods** | **Missing data** | **Statistical significance of findings** |
| Althaus et al.  (2015) |  |  |  |  |  |  |  |  |  |  |  |  |
| Dalton et al.  (2005) |  |  |  |  |  |  |  |  |  |  |  |  |
| Gu et al.  (2015) |  |  |  |  |  |  |  |  |  |  |  |  |
| Van Hecke et al.  (2009) |  |  |  |  |  |  |  |  |  |  |  |  |
| Krach et al.  (2015) |  |  |  |  |  |  |  |  |  |  |  |  |
| Kylliäinen et al. (2012) |  |  |  |  |  |  |  |  |  |  |  |  |

*^Abbreviations: ANS, autonomic nervous system; CNS, central nervous system; ASD, autism spectrum disorder^*
